# Conjectured the Behaviour of a Recycled Metal Matrix Composite (MMC–Al_R_) Developed through Hot Press Forging by Means of 3D FEM Simulation

**DOI:** 10.3390/ma11060958

**Published:** 2018-06-06

**Authors:** Azlan Ahmad, Mohd Amri Lajis, Shazarel Shamsudin, Nur Kamilah Yusuf

**Affiliations:** 1 Department of Mechanical Engineering, Universiti Teknologi PETRONAS, Seri Iskandar 32610, Perak Darul Ridzuan, Malaysia; azlan.ahmad@utp.edu.my; 2 Sustainable Manufacturing and Recycling Technology, Advanced Manufacturing, and Materials Center (SMART-AMMC), Universiti Tun Hussein Onn Malaysia, Parit Raja 86400, Batu Pahat, Johor, Malaysia; shazarel@uthm.edu.my (S.S.); nurkamilah@uthm.edu.my (N.K.Y.)

**Keywords:** sustainable manufacturing, direct metal recycling, hot press forging, aluminium recycling, metal matrix composite, finite element analysis

## Abstract

Melting aluminium waste to produce a secondary bulk material is such an energy-intensive recycling technique that it also indirectly threatens the environment. Hot press forging is introduced as an alternative. Mixing the waste with another substance is a proven practice that enhances the material integrity. To cope with the technology revolution, a finite element is utilised to predict the behaviour without a practical trial. Utilising commercial software, DEFORM 3D, the conjectures were demonstrated scientifically. The flow stress of the material was modified to suit the material used in the actual experiment. It is acknowledged that the stress–strain had gradually increased in each step. Due to the confined forming space, the temperature decreased by ~0.5% because the heat could not simply vacate the area. A reduction of ~10% of the flesh observed in the simulation is roughly the same as in the actual experiment. Above all, the simulation abides by the standards and follows what has been done previously. Through the finite element utilisation, this study forecasted the performance of the recycled composite. The results presented may facilitate improvement of the recycling issue and conserve the environment for a better future.

## 1. Introduction

The improved solid state recycling process is a typical environmentally benign manufacturing process for aluminium alloys. Solid-state recycling, which involves direct recycling of metal scrap into bulk material using severe plastic deformation, has emerged as an alternative to conventional remelting and recycling techniques [1,2,3]. More waste generation was observed than from conventional recycling (melting), and it was also reported that primary aluminium is depleted and other resources should be utilized as alternative materials. Briefly, the hot press forging (HPF) process has been acknowledged as a promising metal forming technique that not only protects the environment but also prevents the generation of new waste [4]. Despite prior evidence that secondary aluminium production could attain superior mechanical properties, it still cannot surpass primary aluminium performance. Therefore, ceramic has been introduced to strengthen the recycled monolithic metal, which is known as a metal matrix composite (MMC) [5]. MMCs have been a subject of scientific investigation and applied research for about two decades but only in the past few years have these advanced materials become realistic candidates in engineering components, such as electronic heat sinks, automotive drive shafts, ground vehicle brake rotors, jet fighter aircraft fins or explosion engine components [6].

Compared to the traditional and competitor processes of metalworking such as the foundry and machining, HPF confers to its manufactured parts excellent mechanical properties thanks to material hardening during deformation and the lack of porosity [7]. In spite of the emergence of new means of manufacturing, the traditional forging process remains advantageous and needs continuous developments, especially in precision forging. This is a liability of using numerical methods that are able to predict the behaviour of materials, but an improvement over the practical hot forging process. The forging process requires various stages of pre-forming operations, each of these stages making it possible to approach the desired final form. These stages require the manufacturing of engravings or the modification of the existing dies. The blacksmith takes some time experimenting before finding an ideal solution. The purpose of the simulation is to not replace the blacksmith in the development of tools and dies but to support and visualize the material flow before any physical fabrication process. To simplify that, the simulation will shorten the trial period and reduce any costs within the trial.

Simulation modelling is the process of creating and analysing a digital prototype of a physical model to predict actual performance [8]. Simulation modelling is used to help designers and engineers understand whether, under what conditions, and in which ways a part could fail and what loads it can withstand. The prediction of material flow can be achieved completely by computer simulation. The main parameters of computer simulation are filling the die completely without leaving any defect, reducing material loss and stress in the die and increasing die life [9]. In order to reduce the cost of the actual forging process and make it competitive with other production methods, it is essential to optimize the design of the part and die [10,11]. Industrial practitioners prefer to implement finite element (FE) modelling before actually executing the design or process in mass production [12,13]. It is a convenient method to design and analyse any new forming processes.

Cavaliere [14] performed finite element (FE) method numerical code to simulate an isothermal forging of the complex component in order to evaluate the stress distribution at a different point. He acknowledged that with the FE method is possible to simulate forming processes and determine the strain, strain rate and damage distribution within the forged parts. With the optimal forging conditions suggested, the component was isothermally forged with a good result in terms of die filling and microstructure. Potnuru [15] deals with the FE analysis of a combined forward and backward extrusion-forging process to produce a socket wrench. It is observed that the flow pattern and forming load required are dependent on the geometry of the product shape, frictional conditions and particularly the die dimension. Aboutalebian [16] conducted normal compression tests using rod specimens, and the tests’ force–stroke curves were compared with the numerical simulation. In their study, they found that there were still small differences between the simulation and experimental results, primarily because of the assumptions and simplifications. Still, there is good agreement between the experimental and simulation results at various solid volume fractions and ram speeds. In addition, researchers have studied the deformation mechanism of forging through adjusting the main parameters while adopting the elastic–plastic dynamic three-dimensional FE method [17,18,19]. Apparently, FE modelling leads to another level of knowledge about the forming behaviour. Nevertheless, the aforementioned works are limited to standard FE parameters, materials, meshing and flow stress. Hence, previous works do not accurately represent the present research.

The present FE study was attempted to investigate the behaviour of a recycled aluminium-based metal matrix composite (MMC–Al_R_) when formed through the HPF process. By manipulating the viscoplastic material model, the property was modified to accommodate the actual aluminium–alumina composite flow stress. The simulation follows the actual HPF process that was previously conducted [20]. The simulations were coupled with the experimental investigation to validate the output through observation from both recorded responses. The main objective was to gain insight into the composite performance in the close-die forging process. To demonstrate the potential of this approach and its suitability for this application, the production of MMC–Al_R_ via the HPF process was investigated through the finite element analysis.

## 2. Methodologies

### 2.1. Materials, Geometries and Meshes

**Figure 1:**
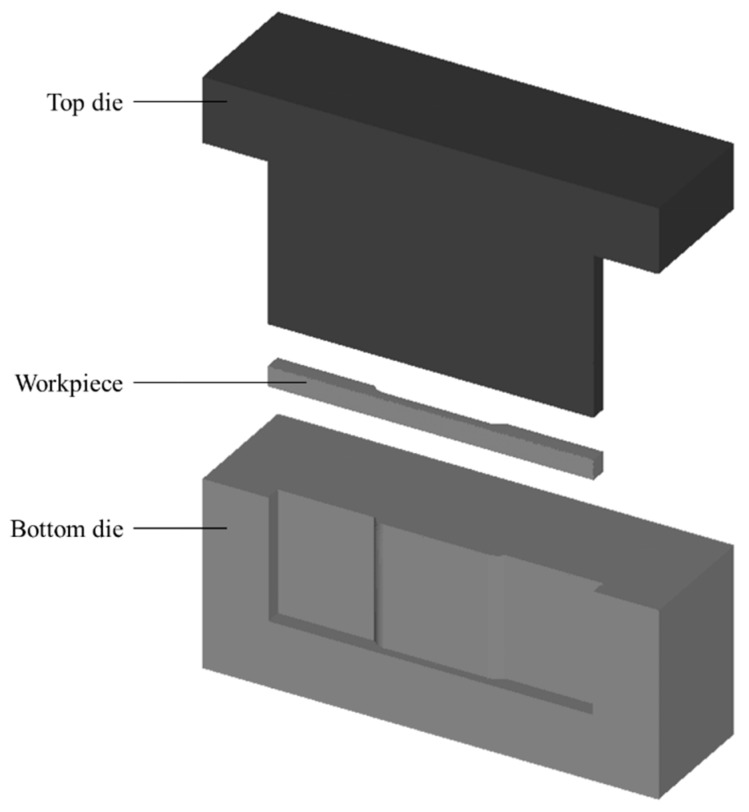
Hot press forging simulation part features drawn from AutoCAD.

Figure 1 depicts the three-dimensional finite element model, which include three parts: (a) top die; (b) workpiece and (c) bottom die. All simulation features were drawn using AutoCAD software and imported into the commercial FE software DEFORM 3D to analyse the proposed forming process. The top and bottom dies are considered thermo-rigid while the workpiece is thermo-viscoplastic, as is the MMC–Al_R_. Hydraulic press is chosen as the movement condition and the plunger is limited to a maximum load of 35 tonnes. Considering the symmetry of the model, a half model was applied to conserve the computational time in the FE simulation. The symmetry planes, which prevent any slippage during the simulation, were declared for all three parts. The correlation of flow stress with strain as a function of temperature and strain rate from the previous literature was utilised [21]. The essential properties for the MMC–Al_R_ material are presented in Table 1.

**Figure 2:**
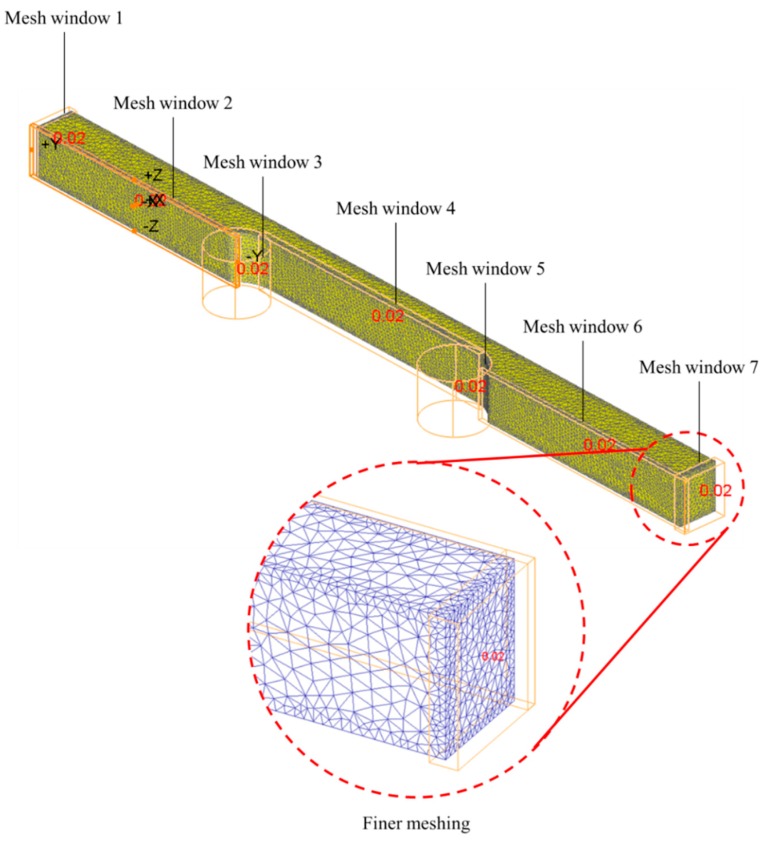
Selective mesh refinement windows.

The meshed within the workpiece domains in DEFORM 3D are generated through the Lagrange algorithm. To obtain a high-quality result without an unnecessarily large time investment during the solving process, selective mesh refinement is applied through the window meshing option. The meshing is applicable for the workpiece part, while the die part remains as is. The meshing for the workpiece employed a tetrahedral element and a total of seven mesh windows were adapted to the workpiece so that it could cope with any uniformity that is present in the critical regions. The seven windows imposed on the workpiece are: (1) two square windows at each end; (2) three rectangular windows on the flat surface; and (3) two circular windows at the shoulder of the gauge, as depicted in Figure 2. The mesh size ratio fixed for all windows was 0.02, which is sufficient to enhance the efficiency of the FE simulation and at the same time obtain high resolution in the areas of particular interest.

### 2.2. Parameters and Boundary Conditions

The process parameter variable, invariably used in the FE analysis, is given in Table 2. The constant shear model was applied to the simulation as part of the boundary conditions. It specifies the workpiece–die surface resistance to metal flow at the interface, as proposed by Orowan in 1943. The constant shear friction model is used mostly for bulk-forming simulations. The frictional force in the constant shear model is defined by

*τ* = *m* × *k*, (1)

where *τ* is the frictional stress, *m* is the friction factor and *k* is the shear yield stress. *τ* is proportional to interface pressure until a critical value is reached. The constant shear frictional model is acknowledged as the popular model since it ostensibly indicated the material features of plastic deformation. The contact interface nodes that have direct contact with the bottom die were selected at the workpiece region. A friction factor of 0.3 was chosen for all contact in accordance with the database for hot forging with a lubricant. The top side of the workpiece will only have the contact node when the top die approaches the surface.

**Figure 3:**
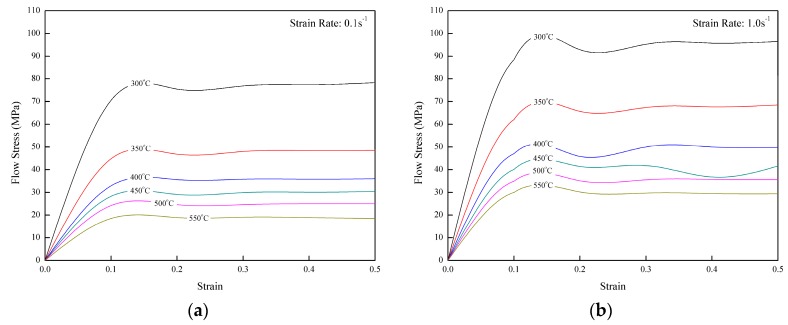
Flow stress–strain curve of homogenized composite 6061-Al_2_O_3_ at strain rate (**a**) 0.1 s^−1^ and (**b**) 1.0 s^−1^.

Figure 3 shows the flow stress–strain curves of a homogenized composite 6061-Al_2_O_3_ at various deformation temperatures and strain rates obtained from the isothermal hot compression tests, as studied by Prasad [21]. The deformation behaviour at eminent temperature is a competitive process between the work hardening and dynamic softening. In the beginning, the flow stress shot up quickly to a certain strain, which is due to the work hardening. As the process proceeded, the flow stress began to settle down and monotonically reached the steady state. This flow stress with two different strain rates (0.1 s^−1^ and 1.0 s^−1^) was logged into DEFORM-3D. Since the strain rate distribution from the actual experiment was 0.25 s^−1^, the software will automatically interpolate the results and come out with the desired strain rate. The interpolation process was also applied for 530 °C (the actual experiment temperature).

### 2.3. Route Overview

**Figure 4:**
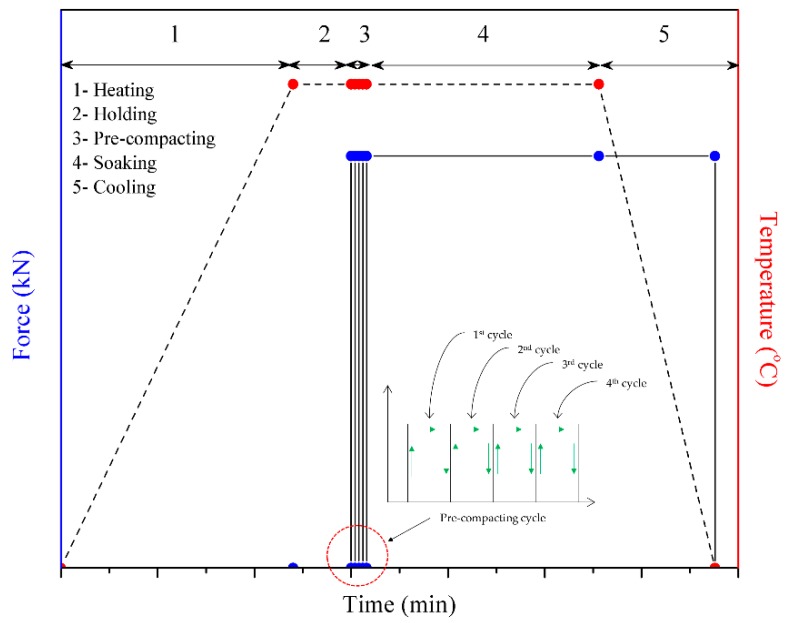
Hot press forging operation flow.

**Figure 5:**
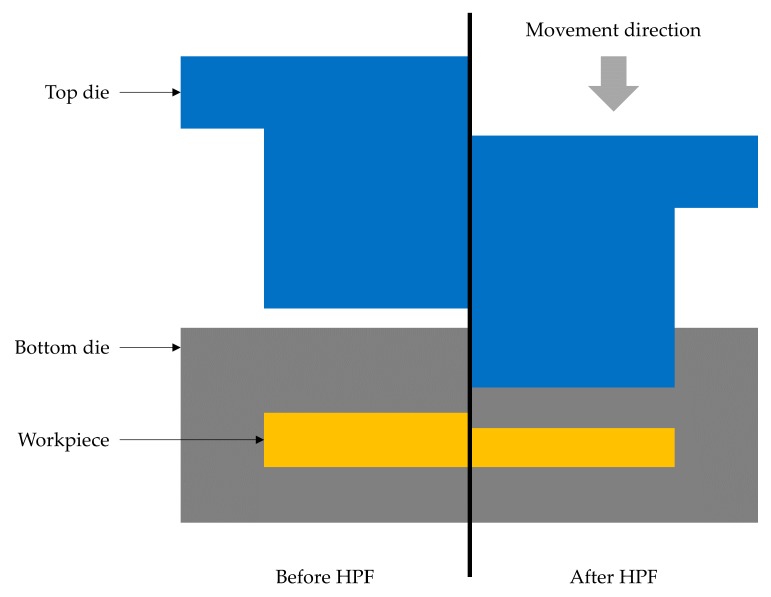
Top die approaching and leaving the workpiece, corresponding to the experimental work.

There are four essential steps involved in the HPF process: (i) holding; (ii) pre-compacting; (iii) soaking and (iv) completion. These stages can be seen in the pressure stage diagram obtained from the FE simulation of HPF, as shown in Figure 4. The stages followed the actual HPF experiment. In the holding stage, the top die does not move, leaving the workpiece load-free. In the experimental work, the holding stage enables the workpiece to gain proper heat conduction within the furnace. This allows the temperature to settle uniformly throughout the die and workpiece. In the second stage, the top die is simulated so that it comes into contact between the die and the workpiece. There were four pre-compacting cycles taking place inside the die. Obviously, the pressure acting on the workpiece is reduced from one cycle to another. This is believed to be caused by the workpiece settlement at high temperature and the high pressure resulting from the previous compacting cycle. Figure 5 shows the top die position and how the workpiece responds before and after the forging cycle.

## 3. Results

The workpiece is subjected to a three-dimensional (3D) state of stress during the deformation process. The stress state is specific to the forming process and dependent on the material behaviour. Prior to the simulation, an introductory investigation was executed to provide preliminary data on the behaviour of the material when it is exposed to a certain force. To obtain an unambiguous result, it is crucial to properly justify the material flow of MMC–Al_R_. The analyses done in the simulation include the effective stress, effective strain, temperature distribution, vector orientation and dimension residuals. The responses observed from the simulation were comparable with the outcome from the actual HPF experiment.

### 3.1. Effective Stress–Effective Strain

**Figure 6:**
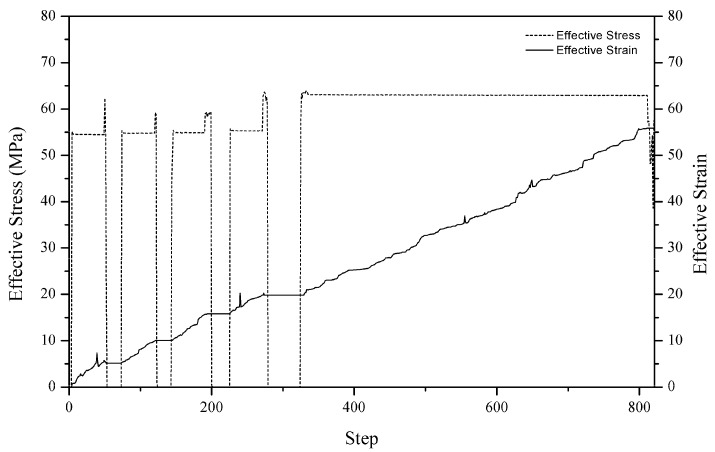
Effective stress–strain recorded for the HPF process at each simulation step.

When the plunger has proximate contact with the workpiece, the pressure acting on the workpiece is parallel to the direction of the movement. The surfaces of the workpiece are mostly affected, particularly in the z-direction. During the forming operation, the workpiece experienced both forging and holding position. Figure 6 depicted the stress–strain distribution throughout the simulation. While having contact with the top die, the workpiece experienced stress and strain. It is to be noted that there was no stress recorded when the plunger was at the holding position. As to the strain, it remains the same when the plunger is on hold. As the cycle moves from one to another, the stress and strain increase. The gradual increase in stress was most probably due to the material behaviour after being pressed to a certain distance.

**Figure 7:**
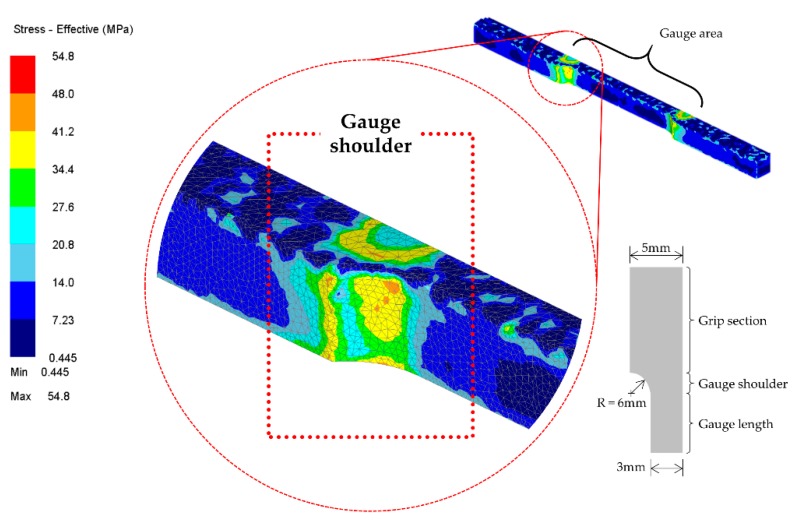
Higher stress recorded at the gauge shoulder.

The stress acting on a material is the force per unit area directed to that material. The force is considered to be uniformly distributed over the acting area, and the reaction force is parallel to the acting vector. The areas play a vital role due to the uniformity of the distribution. Larger surface area resulted in a significant decrease in stress, compared to the smaller surface area. Therefore, a smaller area results in an increase of stress. The findings from the FE modelling are consistent with this theory. The simulated workpiece takes the form of a standard dog bone shape, according to the ASTM-E8 [22], and this was also the shape found in the experiment. Figure 7 illustrated the effective stress recorded by the software. It was highlighted before that the stress response is disproportionate to the acting surface area. Since the gauge surface area is much smaller than that of the other zone, the stresses were predisposed to be concentrated in the gauge area, specifically at the shoulder. Furthermore, the stress recorded at this particular area is higher compared to other regions. Before running the simulation, the element at the gauge areas was predominantly refined through selective mesh refinement to obtain high-quality results. This is due to the earlier hypothesis that these areas are prone to be highly sensitive to stress. Furthermore, after hot forming operation or during the dwell period between each cycle or in the final cooling, the material experiences recovery or recrystallization [23]. As the stages progress to soaking and cooling, the stress is gradually lifted from the gauge area. The stress then appears to be randomly scattered on the workpiece. This is believed to be caused by the compaction activities that were done beforehand. Moreover, the material had reached the equilibrium state after being repeatedly compacted. This made the stress distribute evenly to other regions.

**Figure 8:**
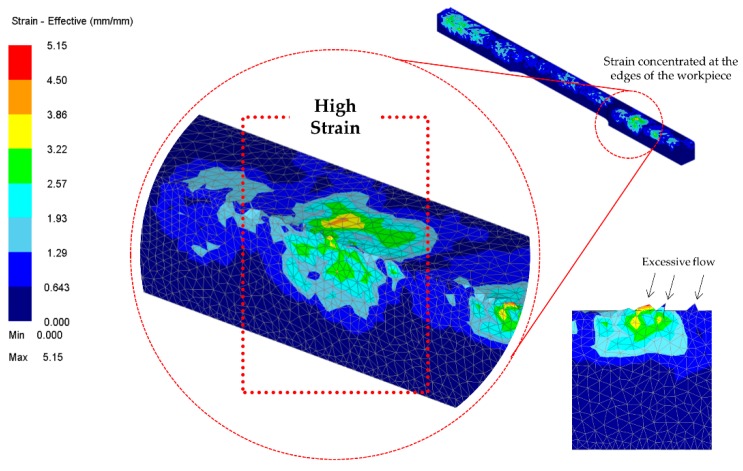
Strain concentration on the edges of the workpiece.

Similarly, the strain stipulates the value of total effective strain at the centroid of each element. Elemental strains are interpolated between meshes during remeshing procedures. Such phenomena may be attributable to the action of the plunger holding on the workpiece. On each cycle, the material is being compacted in a confined space (close-die forging). The material becomes more and more firm, and as the stage progresses, more strain results. In addition, static recrystallization occurs when new nuclei form and grow into new grains at the expense of the deformed material [24]. Such behaviour could be identified from the flesh presence at the workpiece edges. Figure 8 shows the effective strain on the workpiece. It is observed that more strain has accumulated on the edges of the workpiece. Due to the closed-die operation, the only space allotted for the material to flow is between the sharp edges of the die and the plunger. The edges of the fragment were formed due to the flesh from the excessive flow of the material. As the stages progress, the strain is uniformly distributed to the other part of the workpiece.

### 3.2. Temperature Dissemination

**Figure 9:**
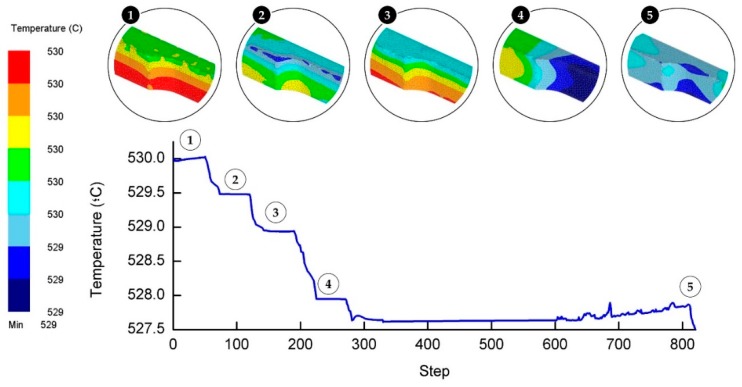
Temperature recorded for HPF process at each simulation step.

The initial temperature of all components was set to 530 °C. By simulating the atmosphere of the actual experiment, the initial temperature is similarly set for all simulation components. Figure 9 depicts the temperature recorded throughout the cycles. The temperature residual all the way through the forming process was calculated to be less than 0.5%. The confined area of close-die forging process was found to attribute to the small decrease in temperature. Heat could not dissipate easily due to the conductive heat transfer that kept the temperature from leaving the die or the workpiece. When working with high temperature, a body of studies should take ample time to achieve the equilibrium thermal state and should stay at a slower pace if the surroundings are also experiencing a higher temperature [25]. When the die comes into contact with the workpiece, the temperature drops slightly. Conversely, the temperature experiences an exponential fall when the die is leaving the workpiece (moving upward) because the opening is exposed to free air. Although the delay from one cycle to another is brief, it contributes to the temperature drop. At the soaking stage, the temperature starts with a monotonic increase and then abruptly rises (steps 600 upward) compared to the other steps. The sudden increase in the temperature is believed to be caused by the prolonged contact time between the die and the workpiece.

**Figure 10:**
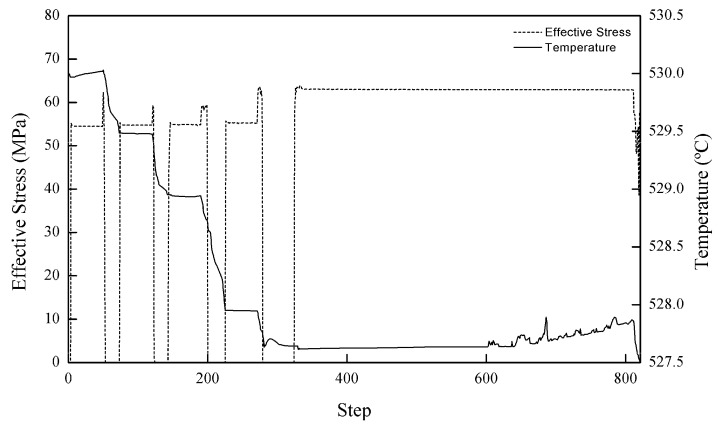
Stress and temperature interaction during the HPF process.

The temperature for all steps was observed to be in line with the concept of conduction heating, as exhibited in Figure 9. In addition, it is clearly illustrated that the temperature at the bottom part is considerably elevated. It is a given that the bottom part is the sole region that has immediate contact with the die. Since the heat cannot evacuate the space, it is maintained with very insignificant residuals. The temperature was slightly higher at the edge of the workpiece as the steps proceed to the other stage. This might be due to the stress imposed when the material is forced to surge through the small aperture between the top and bottom dies. Heat will be more concentrated at the smaller surface and is normally recorded at the burr or ridges of a workpiece while undergoing the shaping or forming process [26]. Figure 10 shows the temperature and stress diagram throughout the HPF simulation. The decrements in temperatures lead to the requirement of higher stress to form the workpiece. A decrease in the temperature means that the material becomes more rigid, and therefore higher stress is needed to deform it.

### 3.3. Vector Orientation

**Figure 11:**
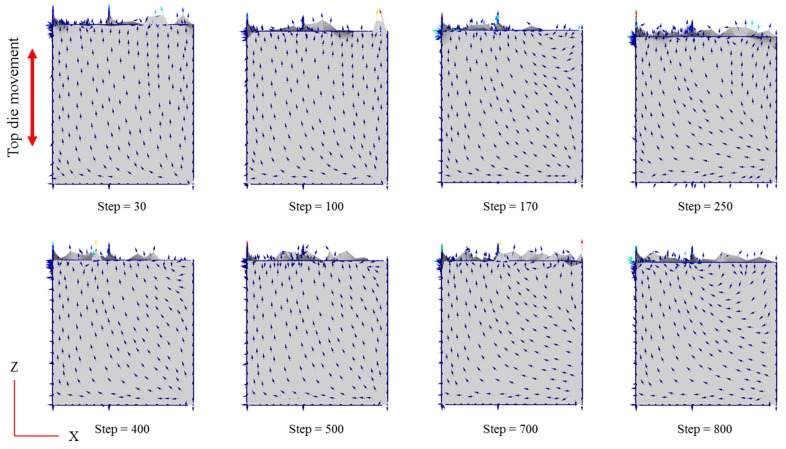
Vector distribution on the workpiece throughout the hot press forging simulation.

Figure 11 shows the velocity fields on the y-axis cross section at selected simulation step corresponding to the tangible HPF experiment. The metal flow inside the confined space is mainly in the z-direction, usually because the top die is approaching in the same direction. Since the forming operation occurs in a confined space, there was movement constriction in both the x-direction and the y-direction.

Additionally, the vectors shift in the x-direction at the bottom of the die throughout all stages. It is believed that, as the metal is forced downward, the bottom region is more able to move freely compared to the other regions. As mentioned previously, the temperature at the bottom part is high because it is the only region where the workpiece has direct contact with the die. These circumstances could also be seen at the side of the workpiece, where the workpiece had direct contact with the die. Subsequently, from the turbulence vector recorded in those particular areas, it can be concluded that a higher temperature leads to high metal flow. The dynamic recovery and local dynamic recrystallization lead to metal softening due to the higher temperature range. At a higher deformation temperature and lower strain rate, geometric dynamic recrystallization occurred and a larger strain resulted in more dynamic recrystallization [27].

Nonetheless, the flesh was reported to experience both vectors in the x-direction and y-direction since it freely moves in either direction. The high pressure of the top die pressing against the workpiece is attributed to the flesh generation, which in turn forces the workpiece to react aimlessly as there is excessive flow at the remaining voids between the top and bottom die.

### 3.4. Dimension Residuals

**Figure 12:**
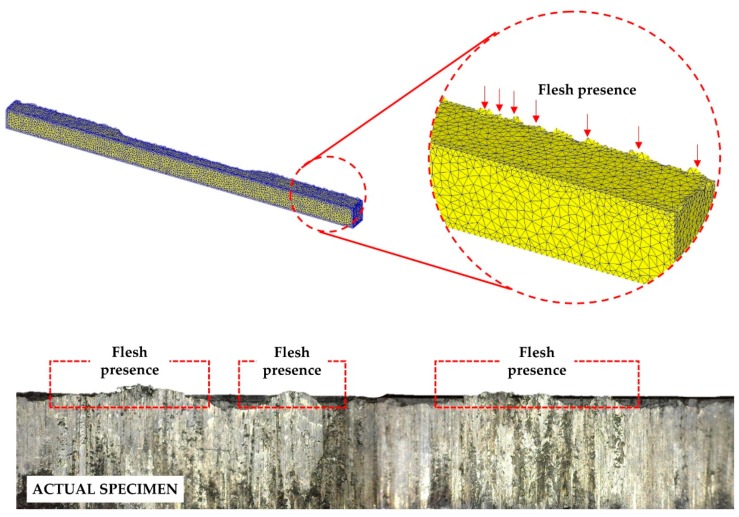
Flesh progression on the workpiece throughout the hot press forging simulation.

Higher stress–strain was identified at the edge of the material, where flesh is likely to be present. Figure 12 shows the workpiece behaviour after the final step of the HPF simulation. The workpiece, which is initially solid, experienced critical deformation throughout the forming operation. As the top die is lowered to form the workpiece, it starts to deform and impairment begins to show. On the 30th step, the workpiece started to be damaged due to the critical value being exceeded. Flesh generation is obviously present on the edges as it is the only region that allows the workpiece access to flow. Since the space between the top and bottom die is diminished, the flesh consequently exists in the form of thin and sharp ridges. The flesh can be simply transformed into an intricate shape caused by the elevated temperature of the top die. The elevated temperature on the top die subsequently reduces the metal flow, thus allowing the metal to slip between the die. When the die stops the operation, the flesh remains on the workpiece. In the last stage, the workpiece solidifies with less damage to the edges. The stages apparently help with condensing the workpiece damage, which yields a better material surface. Furthermore, Figure 12 shows the actual workpiece that resulted from the HPF experiment. The flesh is obviously turned up at the side, where the workpiece has contact with the bottom die.

**Figure 13:**
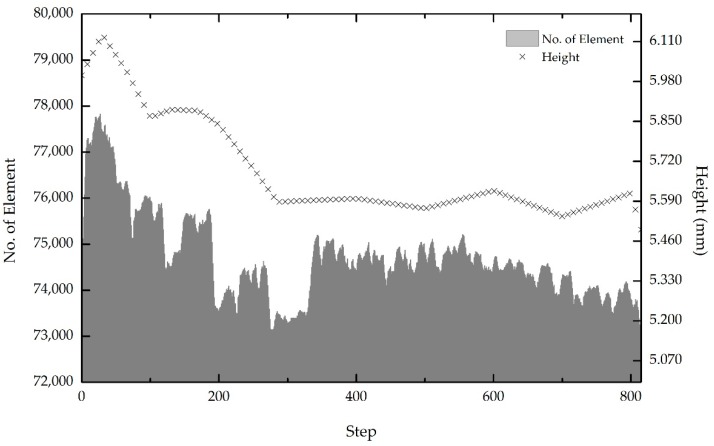
Workpiece height reduction throughout the hot press forging simulation.

The sequential actions of continuous pressing decrease the height of the workpiece flesh, as exhibited in Figure 13. The initial thickness of the workpiece was modelled to be 6 mm. Progressive reduction of approximately 10% of the initial thickness was achieved due to the forging process being assisted by an elevated surrounding temperature. The temperature directly reduces the metal flow and the forging steps, forcing the workpiece to imbue any void within the materials. Similar to in the actual experiment, the voids between the recycled chips are copious. Twelve grams of recycled chips are needed to produce a final product [1,2]. According to the ASTM [22], the thickness of the studied specimen should be less than 6.0 mm. Therefore, both the experiment and simulation are in good agreement with the standard, since the final workpiece height recorded in the simulation is less than 6.0 mm.

Correspondingly, the automated remeshing techniques in the simulation software facilitate precision in data recording. More elements denote comprehensive data acquisition, as fewer residuals are obtained between the areas that are being covered. At the beginning of the simulation, the meshing shapes were coarse. As the stages proceeded, DEFORM 3D automatically recalculated and rebuilt new meshing. This is done to fill the mesh in each of the damaged parts that remain heterogeneous. Figure 13 depicts the element numbers during the forming process. If the initial meshing surpasses the critical value during the operation, the software automatically generates a new mesh that should fill up all areas of concern. This occurs when the materials end up as flesh, burrs or highly damaged during the forming operation. As depicted in the figure, the initial number of elements was set to 74,000. When the forming operation is executed, the meshing is automatically recalculated and the elements are added and then reduced gradually. Additionally, the fluctuation recorded is thought to be caused by the flesh generation. The element for the flesh is very fine and detailed due to it intricate shape. As the cycled proceeded, the number reduced to approximately 72,827 elements. The element numbers fall by roughly 1.58% from the initial amount due to the reduction in thickness. The smaller the area to be covered, the fewer elements are involved in each step.

## 4. Conclusions

Simulation modelling is utilized to better comprehend the behaviour of a material without physically proceeding with the manufacturing process. The material flow can be completely predicted by computer simulation. Through this simulation, the following conclusions are drawn.

- The increase in the effective stress–strain on each step is owing to the proximate contact between the top die and workpiece to deform within the confined space available inside the die. The repeated cycle has resulted in a more rigid workpiece. Therefore, supplementary stress is required to deform the workpiece.
- During the forming process, the temperature distributions were found to be in a delineation manner. Lower forming speed reduces the material temperature and simultaneously increases the forming stress through the operation. The temperature was slightly higher at the edge of the workpiece, which was attributed to the flesh generation.
- The vector orientation of the simulation shows that the workpiece mainly shifts in the z-direction. The side where the workpiece is in contact with the bottom die was recorded to have a relatively robust vector, while the other part of the workpiece maintained its orientation in the z-direction, parallel to the top die movement.
- The flesh generation is deemed to be the main influence on the aggressive stress–strain recording throughout the HPF simulation. However, a comparable form of flesh is observed in the actual HPF sample. This proved that the simulation is in accordance with the experimental response.
- Due to the high stress inflicted on the workpiece and it being kept in a confined space, the thickness is recorded to be reduced by 10%. Contrariwise, the recycled chips in the actual experiment experienced a very large void. The void is concealed during the HPF process, and thus reduces the vertical dimension of the workpiece but still abides by the standard thickness.

## Figures and Tables

**Table 1:** MMC–Al_R_ material properties.

Ultimate tensile strength (MPa)	317.99
Elongation to failure (mm/mm)	20.45
Vickers Hardness (HV)	86.656
Density (g/cc)	2.684
Crystallite Size (Å)	770.6

**Table 2:** Process parameter and frictional condition.

Workpiece length (mm)	100
Workpiece initial thickness (mm)	60
Friction factor at workpiece/die interference	0.3
Reference temperature (°C)	530
Strain and strain rate distribution (s^−1^)	0.250
Minimum size of an element (mm)	0.29
Mesh size ratio	2
Mesh density windows	0.504
Mesh window size ratio	0.02
